# Complete Genome Sequence of Stenotrophomonas maltophilia Phage Philippe

**DOI:** 10.1128/mra.00125-22

**Published:** 2022-05-05

**Authors:** Iris G. Vallavanatt, Mariah Bartz, James Clark, Tram Le, Ben Burrowes, Mei Liu

**Affiliations:** a Department of Biochemistry and Biophysics, Texas A&M University, College Station, Texas, USA; b Center for Phage Technology, Texas A&M University, College Station, Texas, USA; c BB Phage Consultancy, LLC, Georgetown, Texas, USA; Loyola University Chicago

## Abstract

Stenotrophomonas maltophilia is emerging as an opportunistic multidrug-resistant pathogen. S. maltophilia podophage Philippe has a 74,717-bp genome which is related broadly to the N4-like phage group, including *Stenotrophomonas* phage Pokken. The low sequence identity to other described phages suggests that Philippe is an unclassified member of the N4-like subfamily *Rothmandenesvirinae*.

## ANNOUNCEMENT

The ubiquitous, Gram-negative bacterium Stenotrophomonas maltophilia plays an important role in beneficial plant interactions, functioning in both the sulfur and nitrogen cycles and pollutant degradation (1). The emergence of this species as an opportunistic, multidrug-resistant pathogen is a growing concern for immunocompromised patients, and phage therapy is being investigated to address this issue (2, 3). Here, the annotated genome sequence of S. maltophilia podophage Philippe is presented.

Phage Philippe was isolated in January 2019 from a soil sample in College Station, TX (GPS coordinates, 30.60322, −96.36004), using S. maltophilia (ATCC 17807) as the bacterial host. Soil (5 g) was mixed into 10 mL phosphate-buffered saline (PBS) buffer (pH 7.4), and the filtered supernatant (0.2-μm filter) was used for phage isolation. The host was cultured in tryptone nutrient broth (0.5% tryptone, 0.25% yeast extract, 0.1% glucose, 0.85% NaCl, wt/vol) at 30°C with aeration, and phage isolation and propagation were conducted via the soft agar overlay method (4, 5). Genomic DNA was purified from ~8 mL phage lysate using a modified Wizard DNA cleanup kit as previously described (6). DNA sequencing libraries were prepared as 300-bp inserts using a Swift 2S Turbo kit and sequenced on an Illumina MiSeq instrument with paired-end 150-bp reads using v2 300-cycle chemistry. The CPT Galaxy-Apollo phage annotation platform (https://cpt.tamu.edu/galaxy-pub) (7–9) was used for all subsequent analyses except HHpred (10). The 148,548 raw sequencing reads were quality controlled using FastQC (http://www.bioinformatics.babraham.ac.uk/projects/fastqc) and trimmed using the FASTX-Toolkit v0.0.14 (http://hannonlab.cshl.edu/fastx_toolkit/), prior to genome assembly using SPAdes v3.5.0 (11). A single contig was assembled with 94-fold coverage. The contig sequence was completed by PCR amplifying the end region using primers (5′-ATACCCGAGAACAGTGCAGC-3′ and 5′-CTATCTGGATCAGGCTGCCG-3′) and Sanger sequencing the resulting PCR product. Phage termini were predicted using PhageTerm (12). Gene predictions were made using Glimmer v3 (13) and MetaGeneAnnotator v1.0 (14). ARAGORN v2.36 (15) and tRNAscan-SE v2.0 (16) were used to detect tRNA genes. TransTermHP v2.09 was used for the identification of rho-independent termination sites (17). Gene functions were predicted using InterProScan v5.48 (18) and BLAST v2.9.0 (19) against the NCBI nonredundant and Swiss-Prot databases (20). Additional protein analysis was completed using TMHMM v2.0 (21), HHpred (10), LipoP v1.0 (22), and SignalP v5.0 (23). Genome-wide DNA sequence similarity was evaluated using ProgressiveMauve v2.4 (24). All analyses were conducted with default settings. The phage morphology was determined by negatively staining the phage particles with 2% (wt/vol) uranyl acetate and observing them with transmission electron microscopy at the Texas A&M Microscopy and Imaging Center.

**FIG 1:**
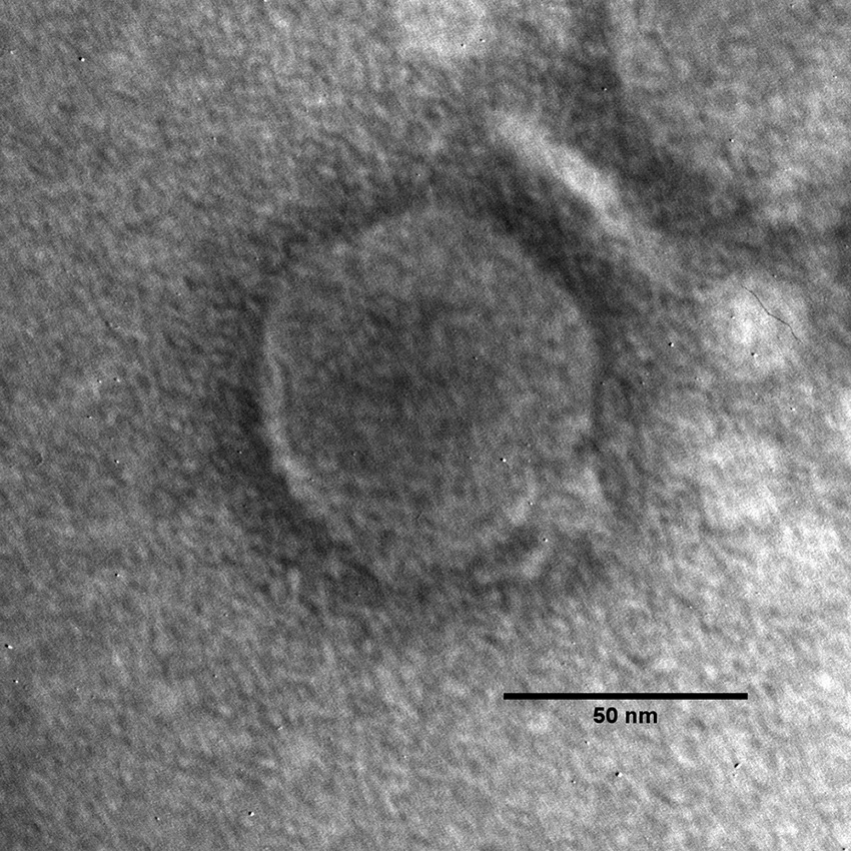
Transmission electron micrograph (TEM) of phage Philippe. Phage particles were diluted with TEM buffer (20 mM NaCl, 10 mM Tris-HCl [pH 7.5], 2 mM MgSO4) and captured on freshly glow-discharged, Formvar carbon-coated grids. The grids were stained with 2% (wt/vol) uranyl acetate and observed on a JEOL 1200 EX TEM instrument at 100 kV accelerating voltage at the Microscopy and Imaging Center at Texas A&M University.

Phage Philippe has a podophage morphology (Fig. 1). It has a 74,717-bp genome with a GC content of 54.3%. A total of 94 protein-coding genes and 6 tRNAs were identified in the Philippe genome. Philippe is related broadly to the N4-like phage group, sharing the highest similarity to *Stenotrophomonas* phage Pokken (GenBank accession number NC_049463), with 26.4% nucleotide identity calculated using ProgressiveMauve and 50 similar proteins (BLASTp; E value, <10^−5^). This low sequence identity to other described phages suggests that Philippe is an unclassified member of the N4-like subfamily *Rothmandenesvirinae*. All components of the lysis cassette in Philippe were identified, including the SAR endolysin *N*-acetylmuramidase, class II holin, and an o-spanin fully embedded within the i-spanin. As expected of an N4-like phage, three RNA polymerase (RNAP) genes were annotated, with the largest identified as the virion RNAP.

### Data availability.

The Philippe genome sequence was deposited in GenBank under accession number MZ326861. The associated BioProject, SRA, and BioSample accession numbers are PRJNA222858, SRR14095251, and SAMN18509471, respectively.

## References

[1] An S-Q, Berg G. 2018. Stenotrophomonas maltophilia. Trends Microbiol 26:637–638. doi:10.1016/j.tim.2018.04.006. 29754971

[2] Brooke JS. 2012. *Stenotrophomonas maltophilia*: an emerging global opportunistic pathogen. Clin Microbiol Rev 25:2–41. doi:10.1128/CMR.00019-11. 22232370 PMC3255966

[3] Adegoke AA, Stenström TA, Okoh AI. 2017. *Stenotrophomonas maltophilia* as an emerging ubiquitous pathogen: looking beyond contemporary antibiotic therapy. Front Microbiol 8:2276. doi:10.3389/fmicb.2017.02276. 29250041 PMC5714879

[4] Adams MH. 1959. Bacteriophages. Interscience Publishers, New York, NY.

[5] van Charante F, Holtappels D, Blasdel B, Burrowes B. 2019. Isolation of bacteriophages. *In* Harper D, Abedon S, Burrowes B, McConville M (ed), Bacteriophages. Springer, Cham, Switzerland. doi:10.1007/978-3-319-40598-8_14-1.

[6] Summer EJ. 2009. Preparation of a phage DNA fragment library for whole genome shotgun sequencing. Methods Mol Biol 502:27–46. doi:10.1007/978-1-60327-565-1_4. 19082550

[7] Ramsey J, Rasche H, Maughmer C, Criscione A, Mijalis E, Liu M, Hu JC, Young R, Gill JJ. 2020. Galaxy and Apollo as a biologist-friendly interface for high-quality cooperative phage genome annotation. PLoS Comput Biol 16:e1008214. doi:10.1371/journal.pcbi.1008214. 33137082 PMC7660901

[8] Afgan E, Baker D, Batut B, van den Beek M, Bouvier D, Cech M, Chilton J, Clements D, Coraor N, Gruning BA, Guerler A, Hillman-Jackson J, Hiltemann S, Jalili V, Rasche H, Soranzo N, Goecks J, Taylor J, Nekrutenko A, Blankenberg D. 2018. The Galaxy platform for accessible, reproducible and collaborative biomedical analyses: 2018 update. Nucleic Acids Res 46:W537–W544. doi:10.1093/nar/gky379. 29790989 PMC6030816

[9] Dunn NA, Unni DR, Diesh C, Munoz-Torres M, Harris NL, Yao E, Rasche H, Holmes IH, Elsik CG, Lewis SE. 2019. Apollo: democratizing genome annotation. PLoS Comput Biol 15:e1006790. doi:10.1371/journal.pcbi.1006790. 30726205 PMC6380598

[10] Zimmermann L, Stephens A, Nam SZ, Rau D, Kubler J, Lozajic M, Gabler F, Soding J, Lupas AN, Alva V. 2018. A completely reimplemented MPI Bioinformatics Toolkit with a new HHpred server at its core. J Mol Biol 430:2237–2243. doi:10.1016/j.jmb.2017.12.007. 29258817

[11] Bankevich A, Nurk S, Antipov D, Gurevich AA, Dvorkin M, Kulikov AS, Lesin VM, Nikolenko SI, Pham S, Prjibelski AD, Pyshkin AV, Sirotkin AV, Vyahhi N, Tesler G, Alekseyev MA, Pevzner PA. 2012. SPAdes: a new genome assembly algorithm and its applications to single-cell sequencing. J Comput Biol 19:455–477. doi:10.1089/cmb.2012.0021. 22506599 PMC3342519

[12] Garneau JR, Depardieu F, Fortier LC, Bikard D, Monot M. 2017. PhageTerm: a tool for fast and accurate determination of phage termini and packaging mechanism using next-generation sequencing data. Sci Rep 7:8292. doi:10.1038/s41598-017-07910-5. 28811656 PMC5557969

[13] Delcher AL, Harmon D, Kasif S, White O, Salzberg SL. 1999. Improved microbial gene identification with GLIMMER. Nucleic Acids Res 27:4636–4641. doi:10.1093/nar/27.23.4636. 10556321 PMC148753

[14] Noguchi H, Taniguchi T, Itoh T. 2008. MetaGeneAnnotator: detecting species-specific patterns of ribosomal binding site for precise gene prediction in anonymous prokaryotic and phage genomes. DNA Res 15:387–396. doi:10.1093/dnares/dsn027. 18940874 PMC2608843

[15] Laslett D, Canback B. 2004. ARAGORN, a program to detect tRNA genes and tmRNA genes in nucleotide sequences. Nucleic Acids Res 32:11–16. doi:10.1093/nar/gkh152. 14704338 PMC373265

[16] Chan PP, Lowe TM. 2019. tRNAscan-SE: searching for tRNA genes in genomic sequences. Methods Mol Biol 1962:1–14. doi:10.1007/978-1-4939-9173-0_1. 31020551 PMC6768409

[17] Kingsford CL, Ayanbule K, Salzberg SL. 2007. Rapid, accurate, computational discovery of Rho-independent transcription terminators illuminates their relationship to DNA uptake. Genome Biol 8:R22. doi:10.1186/gb-2007-8-2-r22. 17313685 PMC1852404

[18] Jones P, Binns D, Chang HY, Fraser M, Li W, McAnulla C, McWilliam H, Maslen J, Mitchell A, Nuka G, Pesseat S, Quinn AF, Sangrador-Vegas A, Scheremetjew M, Yong SY, Lopez R, Hunter S. 2014. InterProScan 5: genome-scale protein function classification. Bioinformatics 30:1236–1240. doi:10.1093/bioinformatics/btu031. 24451626 PMC3998142

[19] Camacho C, Coulouris G, Avagyan V, Ma N, Papadopoulos J, Bealer K, Madden TL. 2009. BLAST+: architecture and applications. BMC Bioinformatics 10:421. doi:10.1186/1471-2105-10-421. 20003500 PMC2803857

[20] The UniProt Consortium. 2018. UniProt: the universal protein knowledgebase. Nucleic Acids Res 46:2699. doi:10.1093/nar/gky092. 29425356 PMC5861450

[21] Krogh A, Larsson B, von Heijne G, Sonnhammer EL. 2001. Predicting transmembrane protein topology with a hidden Markov model: application to complete genomes. J Mol Biol 305:567–580. doi:10.1006/jmbi.2000.4315. 11152613

[22] Juncker AS, Willenbrock H, Von Heijne G, Brunak S, Nielsen H, Krogh A. 2003. Prediction of lipoprotein signal peptides in Gram-negative bacteria. Protein Sci 12:1652–1662. doi:10.1110/ps.0303703. 12876315 PMC2323952

[23] Almagro Armenteros JJ, Tsirigos KD, Sonderby CK, Petersen TN, Winther O, Brunak S, von Heijne G, Nielsen H. 2019. SignalP 5.0 improves signal peptide predictions using deep neural networks. Nat Biotechnol 37:420–423. doi:10.1038/s41587-019-0036-z. 30778233

[24] Darling AE, Mau B, Perna NT. 2010. progressiveMauve: multiple genome alignment with gene gain, loss and rearrangement. PLoS One 5:e11147. doi:10.1371/journal.pone.0011147. 20593022 PMC2892488

